# Evaluation of the Canine Intervertebral Disc Structure in Turbo Spin Echo-T2 and Fast Field Echo-T1 Sequences in Magnetic Resonance Imaging

**DOI:** 10.3389/fvets.2019.00068

**Published:** 2019-03-11

**Authors:** Katarina Kunze, Veronika Maria Stein, Andrea Tipold

**Affiliations:** ^1^Tierarztpraxis Dr. Dieffenbacher, Neustrelitz, Germany; ^2^Vetsuisse Faculty of the University of Veterinary Medicine, Bern, Switzerland; ^3^Department of Small Animal Medicine and Surgery, University of Veterinary Medicine, Hannover, Germany

**Keywords:** intervertebral disc degeneration, Pfirrmann grading system, FFE-sequence, dog, MRI

## Abstract

In the current study the hypothesis should be proven that T1 weighted Fast Field Echo (FFE) sequence is a useful method to visualize intervertebral disc degeneration, respectively changes of the expected disc appearance. Medical records of 208 dogs were reviewed and images of 781 intervertebral discs were evaluated by two blinded examiners using a modified Pfirrmann classification system in two MRI sequences: FFE and Turbo-Spin-Echo T2-weighted sequence (T2W). The patients were allocated to three categories based on body conformation: (1) brachycephalic and chondrodystrophic breeds, (2) non-chondrodystrophic and non-brachycephalic breeds with a body weight of < 25 kg, and (3) non-chondrodystrophic and non-brachycephalic breeds with a body weight greater or equal 25 kg. In brachycephalic and chondrodystrophic dogs 340 intervertebral discs were evaluated, the majority of them presented a mild change of the normal disc structure, 53% in the FFE sequence and 41% in T2W images. High discrepancies were observed between mild and moderate degeneration: in the FFE-sequence 15% (*n* = 50) of the discs had signs of mild degeneration, whereas in T2W the same discs were graded as moderately degenerated. In non-chondrodystrophic and non-brachycephalic breeds under 25 kg body weight 320 intervertebral discs were assessed. In the FFE-sequence 52% (*n* = 166) of the intervertebral discs were judged as having a mild degeneration. In contrast, these same discs were graded as healthy discs (22%), mildly degenerated (33%), moderately degenerated (37%), and severely degenerated (8%) in T2W. In non-chondrodystrophic and non-brachycephalic breeds greater or equal 25 kg 121 intervertebral discs were assessed. The grading was equal in 43%, but differed in one grade (47%) and in two grades (10%) between the two sequences. In both sequences intervertebral disc herniations were equally well-diagnosed. The Kappa coefficient revealed a high discrepancy between the two MRI-sequences. In conclusion, FFE cannot replace the well-established T2W sequence for grading disc degeneration.

## Introduction

Degeneration of the intervertebral disc occurs during physiological aging processes and pathological events (1, 2). Chondroid metaplasia leads to intervertebral disc degeneration in chondrodystrophic breeds early in life (1). In addition, in some brachycephalic breeds, such as french bulldogs, pug dogs, and boxers, morphological changes affecting the vertebral column (spondylosis deformans, thoracical vertebral malformations) are predisposing to intervertebral disc degeneration (3–5). Magnetic resonance imaging (MRI) is currently the gold standard technique to investigate intervertebral disc disease (1, 6, 7). Commonly, for the diagnostic work-up in patients with intervertebral disc herniation and spinal cord compression T2 weighted images (T2W) are evaluated in dorsal, sagittal and transversal planes (8). Currently, T2W images are additionally used to evaluate disc degeneration and can be viewed as gold standard for this evaluation. Further sequences such as pre- and post-contrast T1-weighted (T1W) images, gradient echo sequence or short tau inversion recovery (STIR) are applied to better delineate disc diseases or to rule out paraspinal soft-tissue pathology (9, 10). In T2W images different structures of healthy discs can be distinguished, such as the hyperintense nucleus pulposus surrounded by the annulus fibrosus that is isointense to adjacent muscle tissue (11). Degeneration of the intervertebral disc is a complex, multifactorial process characterized by a decreased signal intensity of the nucleus pulposus and an indistinct border between nucleus pulposus and annulus fibrosus (12, 13). Pfirrmann et al. established 2001 a five stepped grading system focusing on characteristic changes in the structure of the disc on sagittal T2 weighted images (14). Bergknut et al. evaluated and adapted this system for the canine species (15). The gradient echo sequence is another possibility to visualize the morphology of the intervertebral disc with an enhanced T1 or T2^*^ contrast (8). In the literature different terms for gradient echo sequences are mentioned, such as Fast Field Echo (FFE) or Fast Low Angle Short (FLASH) (16). In T1 weighted FFE images, the sequence used in the current study, the intervertebral disc has a homogenous signal intensity isointense to adjacent muscle tissue. In contrast, the vertebral body displays a signal intensity, which is hypointense in comparison to cerebrospinal fluid (8, 17–19). In the present study the hypothesis should be proven that the FFE sequence is a useful method to visualize intervertebral disc degeneration or changes of the expected disc appearance and could give additional information to conventional T2W images, the proposed gold standard. Additionally, we wanted to prove, that both sequences are capable to detect disc herniation. To verify these hypotheses, more than 700 single intervertebral discs were evaluated by two blinded examiners.

## Materials and Methods

### Ethics Statement

The study was conducted following the guidelines of the University of Veterinary Medicine Hannover with written consent of the dog owners and approved by the thesis committee of the University.

### Case Selection

Medical records of 208 dogs presented between 2010 and 2012 at the Dept. of Small Animal Medicine and Surgery, University of Veterinary Medicine Hannover, with different neurological disorders were retrospectively reviewed. Dogs were included, when radiographs and MR images of the vertebral column were available. According to their body conformation the patients were allocated to three categories. Category 1 included brachycephalic and chondrodystrophic breeds. Category 2 comprised non-chondrodystrophic and non-brachycephalic breeds with a body weight < 25 kg and category 3 encompassed non-chondrodystrophic and non-brachycephalic breeds with a body weight equal or greater 25 kg. Breeds were classified as chondrodystrophic or brachycephalic according to the description of Parker et al. (20) and Bannasch et al. (21)

### Magnetic Resonance Imaging

**Figure 1:**
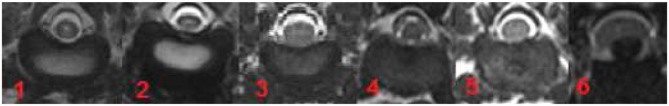
Grading system for T2W images modified after Pfirrmann et al. (14). Grade 1 and 2 included healthy intervertebral discs in dogs younger (grade 1) or older than 12 months (grade 2). Nucleus pulposus is hyper- or isointense to cerebrospinal fluid with a clear distinction between nucleus pulposus and annulus fibrosus. Grade 3 is characterized by mild degeneration affecting at the maximum one-third of the intervertebral disc with a iso- or hypointense nucleus pulposus and a visible distinction between nucleus pulposus and annulus fibrosus. Moderate degeneration (grade 4) affected at the minimum one-third and at the maximum two-third of the intervertebral disc, the distinction between nucleus pulposus and annulus fibrosus is blurred. In severe degeneration (grade 5) more than two-third of the intervertebral disc is affected, the nucleus pulposus is hypointense, narrowing of the cerebrospinal fluid space is possible. Disc herniations are displayed under “6”.

**Figure 2:**
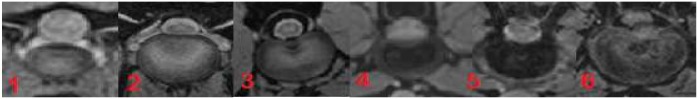
Grading system for T1W FFE-sequence (designed by KK and AT). Grade 1 and 2 included healthy intervertebral discs in dogs younger (grade 1) or older than 12 months (grade 2). The disc displays a homogeneous structure and a signal intensity isointense to adjacent muscles (Mm trunci). Grade 3 is characterized by mild degeneration affecting at the maximum one-third of the intervertebral disc with a hypointense nucleus pulposus. Moderate degeneration (grade 4) affected at the minimum one-third and at the maximum two-third of the intervertebral disc (hypointense to surrounding tissue). In high grade degeneration (grade 5) more than two-third of the intervertebral disc is affected and hypointense to the surrounding tissue. Disc herniations are displayed under “6”.

MRI was performed using a 3-Tesla MRI scanner (Philips Achieva, Eindhoven, The Netherlands). The intervertebral discs were evaluated in the transverse plane using a modified Pfirrmann classification system in Turbo-Spin-Echo T2-weighted sequence (T2W) (TR = 3100 ms, TE = 120 ms, flip angle = 90°) and a grading system designed by the authors in T1 weighted FFE-sequence images (FFE) (TR = 11 ms, TE = 220 ms, flip angle = 8°). MR images were reviewed by two board-certified neurologists (AT and VMS) blinded to signalement and clinical data. Five grades of disc degeneration were used to evaluate the appearance and potential degeneration of the intervertebral discs (Figures 1, 2). Disc herniation was defined as “6”, independent of the status of degenerative changes. In the current study “6” does not reflect the degree of disc degeneration.

### Statistics

Analyses were assessed with the statistical software SAS, version 9.2 (SAS Institute, Cary, NC, USA). For comparison of the two MRI-sequences McNemar's test was used and the simple and weighted kappa coefficient established.

## Results

A total of 208 patients were examined. Eighty-three dogs were assigned to category 1, 86 patients to category 2, and 39 patients to category 3. In category 1, 63.9% (*n* = 53) were Dachshunds and 19.3% (*n* = 16) French bulldogs. Further breeds in category 1 included Pug dogs (*n* = 4), Boxers (*n* = 3), Shi-Tzu (*n* = 3), Havanese (*n* = 2), Sky terrier (*n* = 1) and Welsh corgi cardigan (*n* = 1). The category 2 included 27 breeds, mainly Beagles (*n* = 11), Jack Russell terriers (*n* = 10) and mixed breed dogs (*n* = 23). In category 3 a total of 17 breeds were presented, especially German Shepherd dogs (*n* = 8), Labrador Retrievers (*n* = 7), and mixed breed dogs (*n* = 9). 45 males (7 neutered) and 38 females (23 spayed) were included in category 1, 59 males (14 neutered) and 27 females (19 spayed) in category 2 and 25 males (6 neutered) and 14 females (6 spayed) in category 3. Bodyweight ranged from 3.9 to 29.8 kg (median 8.1 kg) in category 1, from 2.6 to 24 kg (median 11 kg) in category 2, and from 25 to 60 kg (median 34 kg) in category 3. The youngest patients of the study were < one 1 year old and the oldest patients were 14 years old. The mean age of dogs suffering a disc extrusion was 5.73 years in category 1, 6.67 years in category 2, and 7.44 years in category 3.

A total of 781 intervertebral discs was evaluated in T2W and in T1 weighted FFE-sequence, which included 340 in category 1, 320 in category 2, and 121 in category 3 of dogs. 21 % (*n* = 164) of the intervertebral discs were located between C2 and T1, 44.7% (*n* = 349) between T1 and L1, and 34.3% (*n* = 268) between L1 and the sacrum. AT and VMS evaluated together the structure of the intervertebral discs using the described grading system for MR images and were blinded regarding signalement and clinical signs. Grades were ascertained after achieving a consensus between the two examiners. Evaluating the images using the described grading system statistically significant differences were found between the two MRI sequences in all three categories of dogs (*p* < 0.0001 in category 1 and 2, *p* = 0.001 in category 3). The results of the simple and weighted Kappa coefficient confirmed these results (Table 1).

**Table 1:** Results of kappa coefficient in three categories of dogs.

	**Category 1**	**Category 2**	**Category 3**
Simple Kappa coefficient	0.5703	0.3844	0.2860
Weighted Kappa coefficient	0.7779	0.6547	0.5996

The Kappa coefficient was chosen to demonstrate agreement between evaluation of the different grades of intervertebral disc degeneration by using the grading system for each sequence. In category 1 of dogs the highest agreement compared to the other categories was present. The higher score of the weighted Kappa coefficient reflected the finding that the evaluation of morphologic disc appearance deviated in the majority by one grade.

In both sequences the same number of intervertebral disc extrusions (“6”) was assessed.

**Figure 3:**
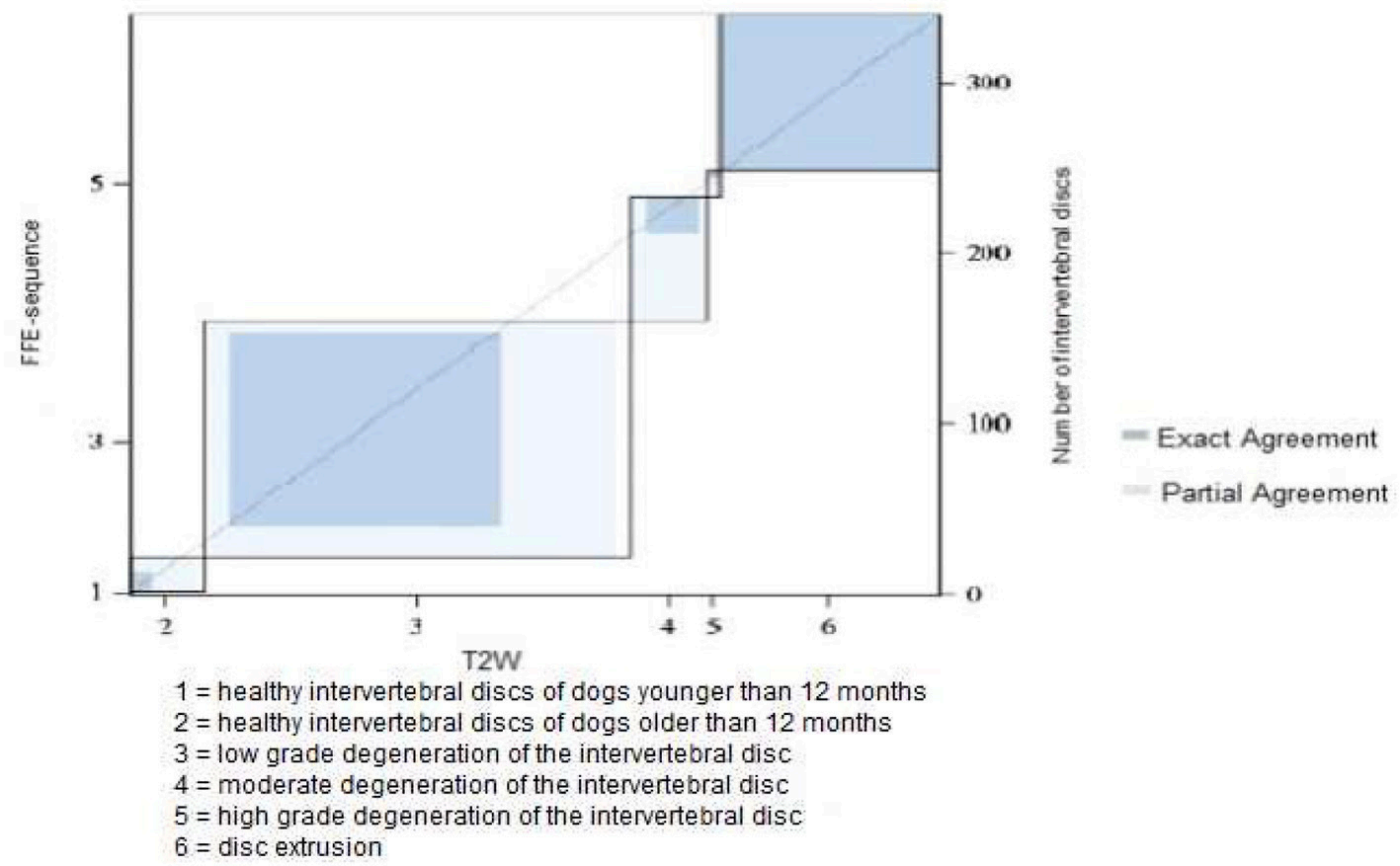
Results of grading system agreement for intervertebral discs between T1 weighted FFE-sequence and T2 weighted images in brachycephalic and chondrodystrophic breeds (category 1).

In category 1 of dogs a total of 340 intervertebral discs were evaluated. The majority of the discs were judged to have mild degeneration, 53% in the FFE-sequence and 41% in T2 weighted images. A high discrepancy was observed between evaluating mild or moderate grade degeneration. 15% (*n* = 50) of the discs were considered to have mild degeneration in the FFE-sequence. In T2 weighted images the same discs were assessed as moderate grade degeneration. The mean value of the grading was 3.84 in the FFE-sequence and 4.04 in T2W for category 1 of dogs. In T2 weighted images signs of disc degeneration were graded on average higher than in the FFE-sequence. In total an exact agreement between disc assessment in FFE and T2W sequences was made in 240 discs (71%). The evaluation of ninety discs (26%) differed in one grade and a partial agreement was achieved (Figure 3). No agreement was seen in 10 discs (3%) and the evaluation differed in more than one grade.

**Figure 4:**
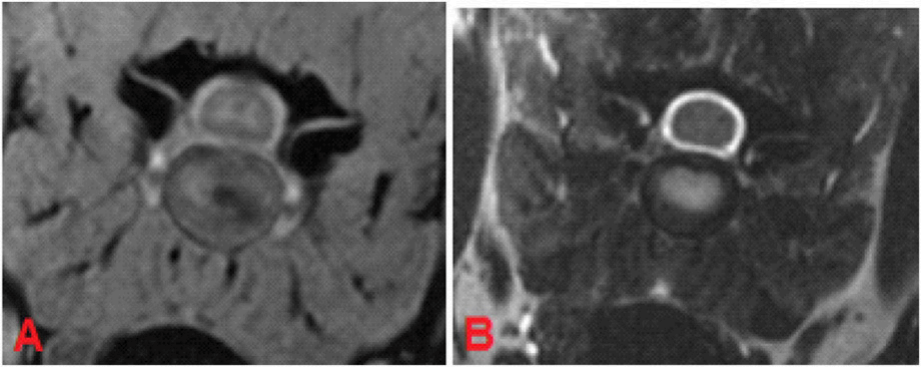
MRI of C5-C6 intervertebral disc, transverse plane, patient 88. In the FFE-sequence a central area of decreased signal intensity in the nucleus pulposus was present accounting for less than one-third of the intervertebral disc and was evaluated as grade 3 **(A)**. In T2W the intervertebral disc was composed of a hyperintense nucleus pulposus and hypointense annulus fibrosus and was judged to be a healthy disc from an adult dog, grade 2 **(B)**.

In category 2 of dogs the evaluation of the intervertebral discs revealed a higher grading in T2W images. On average the mean value of the grading was 3.78 in FFE-sequence and 4.09 in T2W images. The presence of mild degeneration was assessed differently between the both sequences. 52 % (*n* = 166) of the intervertebral discs showed signs of mild degeneration in the FFE-sequence. In T2W images only 23% (*n* = 75) presented these findings. Nine percent (*n* = 30) of the discs were graded “healthy” in T2W images, but showed signs of mild degeneration in the FFE-sequence (Figures 4A,B).

**Figure 5:**
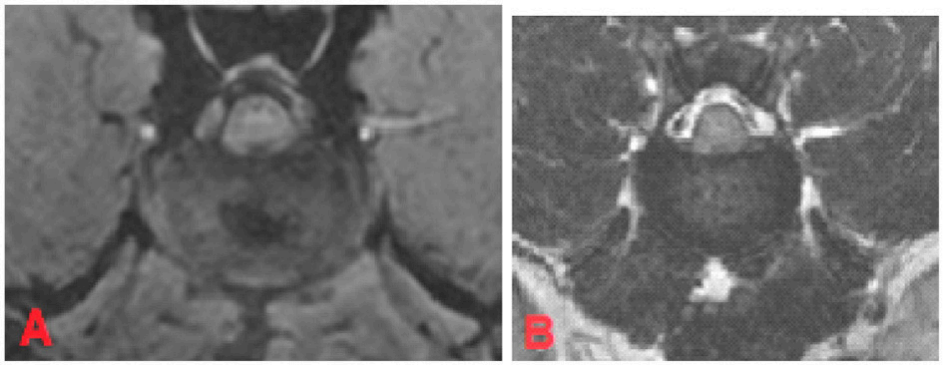
MRI of the L1-L2 intervertebral disc, transverse plane, patient 111. In FEE-sequence the intervertebral disc had an inhomogeneous structure with a central hypointense region which takes ~18% of the intervertebral disc and was judged as “mild degeneration” **(A)**. In T2W there was an unclear distinction between nucleus pulposus and annulus fibrosus. The signal intensity of the nucleus pulposus was hypointense to cerebrospinal fluid. The disc was assessed to have “moderate degeneration” **(B)**.

On the other hand, 19.1% (*n* = 61) of the intervertebral discs were classified as having “mild degeneration” in the FFE-sequence, whereas in T2W images signs of moderate grade degeneration were visible (Figures 5A,B).

**Figure 6:**
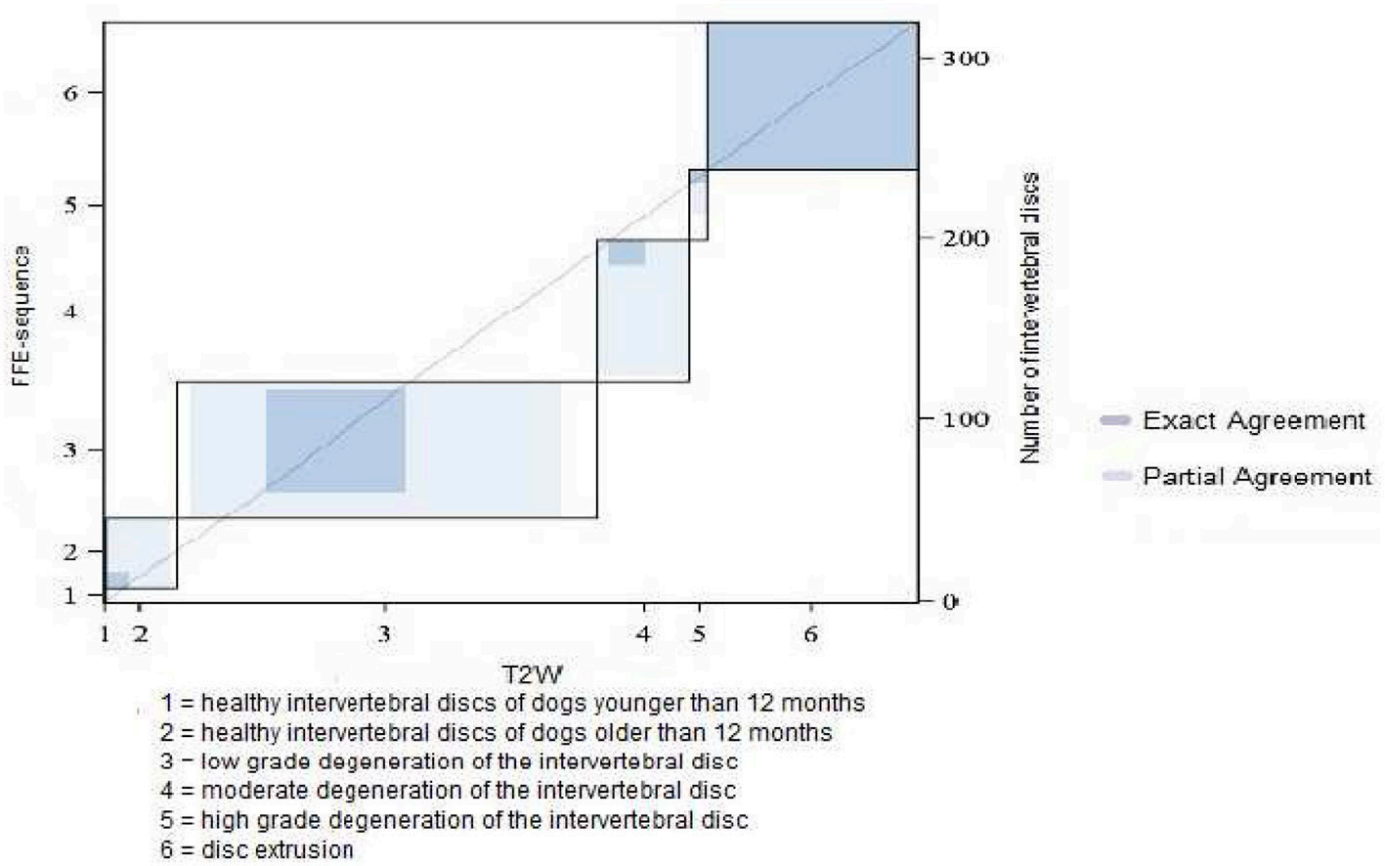
Results of grading system agreement for intervertebral discs between T1 weighted FFE-sequence and T2 weighted images in non-chondrodystrophic and non-brachycephalic breeds with a body weight under 25 kg (category 2).

The evaluation of the intervertebral discs was identical in 52.2% (*n* = 168) in both sequences, a partial agreement was achieved in 40.3% (*n* = 129) of the intervertebral discs (Figure 6).

**Figure 7:**
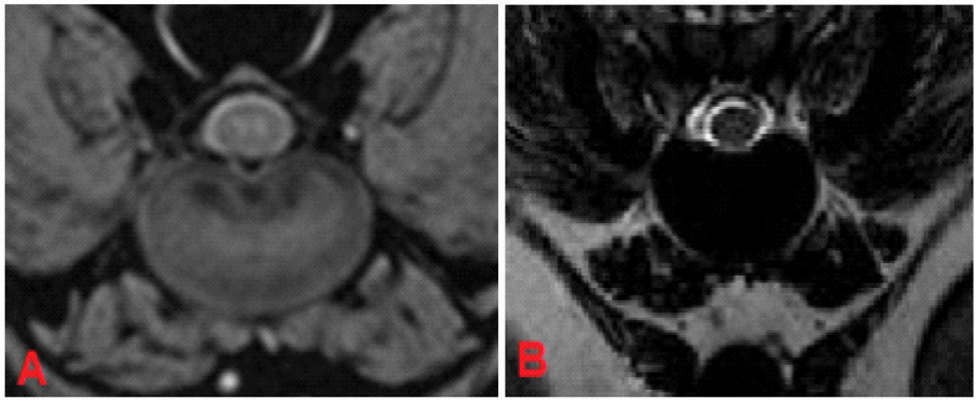
MRI, T13-L1 intervertebral disc, transverse plane, patient 175. In FFE-sequence small hypointense areas were present leading to the assumption of a low grade degeneration **(A)**, whereas in T2W the intervertebral disc displayed a complete signal loss without any possibility to differentiate between nucleus pulposus and annulus fibrosus, grade 5 **(B)**.

**Figure 8:**
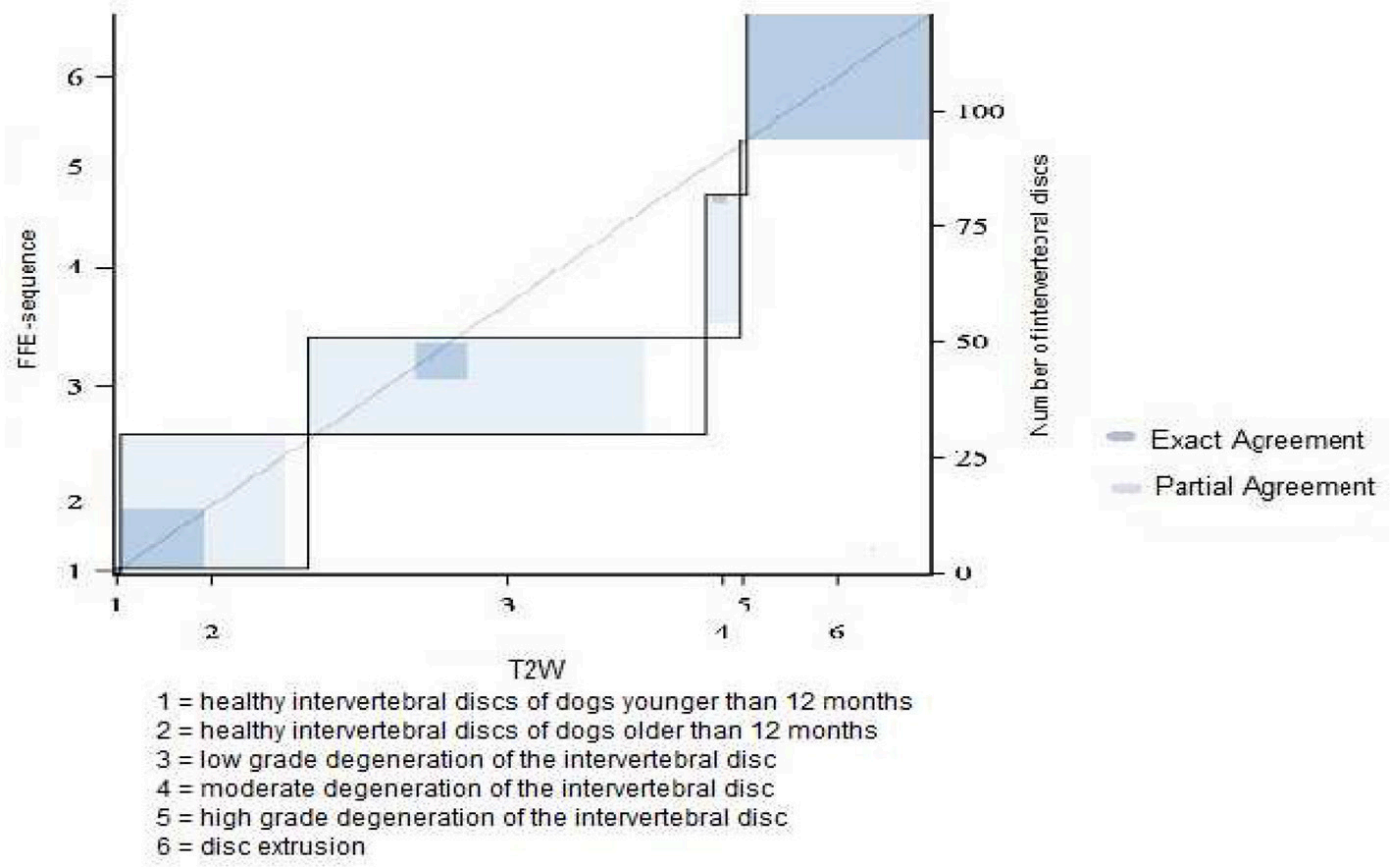
Results of grading system agreement for intervertebral discs between T1 weighted FFE-sequence and T2 weighted images in non-chondrodystrophic and non-brachycephalic breeds with a body weight greater or equal 25 kg.

In category 3 of dogs 121 intervertebral discs were evaluated. The average grade was lower than in other dog breeds: 3.48 in FFE-sequence and 3.87 in T2W. The assessement was equal in 43% (*n* = 52) of the intervertebral discs and differed in one grade in 47% (*n* = 57) and in two grades in 10% (*n* = 12) of the intervertebral discs. (Figures 7A,B, 8)

In addition, the capability of the two sequences to detect disc herniation should be assessed as the second aim of the study. Intervertebral disc herniations occurred in 22 intervertebral discs in category 1, 38 intervertebral discs in category 2, and 17 intervertebral discs in category 3 of patients. In both sequences disc herniations could be equally well-diagnosed.

## Discussion

Intervertebral disc degeneration is a common pathological finding in dogs and can predispose dogs to disc extrusion (22). However, not every intervertebral disc degeneration lead to herniation, spinal cord compression and to neurological deficits such as ataxia or paraparesis/paraplegia (22, 23). MRI (T2W sequence) is considered to be the gold standard to identify intervertebral disc degeneration *in vivo* (1, 6, 24). For the classification of the extent of intervertebral disc degeneration the Pfirrmann classification system was used and evaluated for the dog by Bergknut et al. (15) In this classification system, the intervertebral disc is evaluated in T2 weighted images and this sequence can currently be considered as gold standard. The normal nucleus pulposus appears hyperintense in comparison to surrounding tissue and correlating to proteoglycan concentrations (10, 14). Modic et al. (25) confirmed that the decrease of the signal intensity in T2W correlates with the progression of intervertebral disc degeneration. Kranenburg et al. (6) could conclude that intervertebral disc degeneration did not correlate with the severity of neurological signs. In the current study, the Pfirrmann classification system for the evaluation of intervertebral discs was used in T2 weighted images with slight modifications. For the grades 3, 4, and 5 the percentage of the extent of intervertebral disc degeneration was added. In addition, the evaluation was performed in transverse planes. This sequence was compared to a different technique, the T1W FFE-sequence in order to evaluate the suitability of this sequence in detecting structural disc changes. However, the hypothesis could not be proven that the FFE sequence is an equally useful method to visualize intervertebral disc degeneration. In the current study, it could not be evaluated, if these structural changes were indeed degenerative changes, since histopathological examinations were not performed. In comparison to T2W sequence the intervertebral disc displayed a more homogeneous structure without clear distinction between nucleus pulposus and annulus fibrosus in T1W FFE sequence as already described (18, 19). Despite this more homogenous structure grades 3, 4, and 5 could be well-defined and even the percentage of potential structural changes of the disc could be evaluated.

Statistically significant differences were found between both MRI-sequences evaluating intervertebral disc degeneration, respectively changes of the expected MRI appearance, whereas intervertebral disc extrusions and protrusions were equally diagnosed by the two sequences. The presence of mild degeneration was diagnosed differently in both sequences. In T1 weighted FFE-sequence mild degeneration was seen more frequently than in T2W images, 53% of intervertebral discs in the first category of dogs, 52% of the intervertebral discs in the second category and 49% of the intervertebral discs in the third category showed signs of mild degeneration. In contrast, in T2 weighted images only 41% of the intervertebral discs in category 1, 23% of the intervertebral discs in category 2, and only 17% of the intervertebral discs in category 3 displayed these signs. The evaluation of discs with mild degeneration in the T1 weighted FFE-sequence ranged from a healthy disc to a severely degenerated disc in T2 weighted images: a healthy disc in T2 weighted images was judged in 47% in category 1 of dogs, 77% in category 2, and in 55% in category 3 signs of mild degeneration in the T1 weighted FFE-sequence, suggesting that slight changes are only visible in the T1 weighted FFE-sequence or that this sequence is more susceptible to artifacts. To prove that the described morphological changes in the T1 weighted FFE-sequence is indeed a pathological finding studies have to be performed in the future comparing MRI findings and histopathology. Interestingly, when degenerative signs were clearly visible in T2W images, signs of disc degeneration were graded on average higher than in the T1 weighted FFE-sequence. In addition, the FFE-sequence was vulnerable to susceptibility artifacts because of the absence of the 180° refocusing pulse (8). In conclusion, the present study demonstrated that T1 weighted FFE sequence is not a precise alternative to T2W images and the discrepancies between the two sequences have to be further evaluated. The superiority or inferiority of T1 weighted FFE has still to be proven to achieve an additional method to diagnose intervertebral disc degeneration. In the current study, it was not confirmed, that the described abnormal appearance of discs in T1W FFE sequence is indeed a degenerative change. A limitation of the study is, that histological examinations of the discs were not performed. All discs evaluated with the T1W FFE sequence were compared to the already well-described appearance in T2W sequence supporting the suggestion that a degeneration is highly probable. However, slight changes of the intervertebral disc structure are better visible in T1 weighted FFE images and may only represent intrinsic sequence changes. Therefore, future studies in dogs undergoing necropsy are recommended to evaluate this phenomenon.

## Author Contributions

KK performed the study and drafted the manuscript. VS evaluated MR images and revised the manuscript. AT designed the study, evaluated MR images and revised the manuscript.

### Conflict of Interest Statement

The authors declare that the research was conducted in the absence of any commercial or financial relationships that could be construed as a potential conflict of interest.
